# Plasma‐activated medium triggers immunomodulation and autophagic activity for periodontal regeneration

**DOI:** 10.1002/btm2.10528

**Published:** 2023-05-04

**Authors:** Shengfang Wang, Peiyu Wang, Rik Thompson, Kostya Ostrikov, Yin Xiao, Yinghong Zhou

**Affiliations:** ^1^ Centre for Biomedical Technologies Queensland University of Technology Brisbane Queensland Australia; ^2^ State Key Laboratory of Cellular Stress Biology, School of Life Science Xiamen University Xiamen China; ^3^ School of Biomedical Sciences Queensland University of Technology Brisbane Queensland Australia; ^4^ Translational Research Institute Woolloongabba Queensland Australia; ^5^ State Key Laboratory of Molecular Vaccinology and Molecular Diagnostics & Center for Molecular Imaging and Translational Medicine, School of Public Health Xiamen University Xiamen China; ^6^ Centre for Materials Science Queensland University of Technology Brisbane Queensland Australia; ^7^ School of Chemistry and Physics, Faculty of Science Queensland University of Technology Brisbane Queensland Australia; ^8^ School of Medicine and Dentistry Griffith University Gold Coast Queensland Australia; ^9^ School of Dentistry, Faculty of Health and Behavioural Sciences The University of Queensland Brisbane Queensland Australia

**Keywords:** autophagic activity, immunoregulation, periodontal regeneration, plasma‐activated medium (PAM)

## Abstract

Periodontitis is an infection‐induced inflammation, evidenced by an increase in inflammatory macrophage infiltration. Recent research has highlighted the role of plasma‐activated medium (PAM) as a regulator of the innate immune system, where macrophages are the main effector cells. This study therefore aims to investigate the immunomodulatory effects of PAM on macrophages and its potential applications for periodontitis management. PAM was generated using an argon jet and applied to culture macrophages. Proinflammatory macrophage markers were significantly reduced after PAM stimulation, and this was correlated with the activation of autophagy via the Akt signaling pathway. Further investigations on the proregenerative effects of PAM‐treated macrophages on periodontal ligament cells (PDLCs) revealed a significant increase in the expression of osteogeneis/cementogenesis‐associated markers as well as mineralization nodule formation. Our findings suggest that PAM is an excellent candidate for periodontal therapeutic applications.

## 1 INTRODUCTION

As an essential component of the innate immune system, macrophages protect against harmful pathogenic microorganisms and regulate immune responses.^1^ This is accomplished by both the classically activated M1 subtype and the alternatively activated M2 subtype.^1^, ^2^ The proinflammatory functions of M1 macrophages include phagocytosing pathogens or foreign particles, presenting antigens to other immune cells, and secreting inflammatory cytokines to trigger the inflammation.^3^ However, M1 macrophages would cause chronic inflammation and damage the tissues if they continuously released cytotoxic substances into the microenvironment.^4^ Anti‐inflammatory cytokines produced by M2 macrophages, on the other hand, would encourage tissue regeneration.^1^ Periodontitis, a common bacterial infection‐induced chronic inflammation, often results in tooth loss leading to major aesthetic and functional issues if left untreated.^5^ Excessive M1 macrophages and uncontrolled M1 macrophage‐mediated cytokine secretion are the significant factors that cause the destruction of periodontium, including periodontal ligament (PDL), cementum and alveolar bone.^4^ Therefore, restoring the balance between M1 and M2 macrophages is essential for periodontitis patients to reduce chronic inflammation and enhance tissue regeneration.

Plasma is an important source of charged particles, free radicals, electric fields, and metastable species.^6^ Cold atmospheric plasma (CAP) has shown great promise in treating conditions such as cancer, skin infections, and blood clotting.^7^, ^8^, ^9^, ^10^, ^11^ In clinical research of oral cancers and infections, CAP has been applied orally without safety concerns,^12^, ^13^, ^14^ suggesting that CAP is safe to be used as a therapeutic option. Since it is challenging to deliver CAP directly to the target in vivo, CAP activity is often converted to liquid, providing a versatile method for therapeutic application.^10^, ^11^ One plasma‐activated liquid that exhibits therapeutic properties and has recently been explored, is plasma‐activated medium (PAM).^10^, ^15^ PAM produced from CAP‐activated medium is cytotoxic to cancer cells but has no discernible impact on normal cells.^10^ One notable advantage of PAM compared to CAP is its ability to access oral cavities and penetrate deep tissues which CAP cannot. Additionally, PAM is considered safer for cell treatment as it does not retain components such as ultraviolet rays and electromagnetic fields. Moreover, the effective treatment time for PAM treatment (hour level) is longer than that for CAP maintenance (minute level). Therefore, to promote the practical application of cold plasma technology in clinical settings, PAM is utilized as a versatile CAP method in this study.

Our previous studies^15^, ^16^, ^17^ demonstrated that PAM is distinct from individual ROS/RNS compound solutions. Through secondary reactions, PAM can continuously generate nanosecond and microsecond short‐lived free radicals through secondary reactions, such as hydroxyl radicals (·OH), singlet oxygen (^1^O_2_), and peroxynitrite anions (ONOO^−^), when applied to biological tissues. PAM can also act as an inducer in the innate immune system, according to previous studies.^18^, ^19^ However, more research is needed to determine whether PAM has the potential to promote periodontal regeneration by modulating macrophage responses. This project therefore investigated the immunomodulatory effect of PAM on macrophages and its application as a therapeutic approach for periodontitis.

## 2 MATERIALS AND METHODS

### 2.1 PAM generation

A conventional commercial argon plasma jet (model kINPen 09, INP Greifswald, Germany) was used in this study to generate CAP.^20^ Aliquots of 1.5 mL serum‐free medium (Dulbecco's Modified Eagle Medium, DMEM; Life Technologies, USA) containing 50 U/mL penicillin and 50 μg/mL streptomycin (P/S; Life Technologies Australia Pty Ltd.) was activated by CAP for 10 min at a flow rate of 5.0 Standard Liter per Minute while being exposed to pure argon gas. The plasma jet nozzle was 10 mm away from the medium surface. Serum‐free DMEM containing 50 U/mL penicillin and 50 μg/mL streptomycin was used to dilute PAM to various concentrations (5%, 10%, and 30%). Optical emission spectroscopy (OES), SpectraPro‐750i monochromator (Acton Research Corporation, USA), was used to identify several reactive atoms and molecules found in PAM.^11^

### 2.2 Macrophage culture stimulation

The murine macrophage cell line RAW264.7 was grown in DMEM with 1% P/S, 10% fetal bovine serum (FBS) at a density of 5000 cells per cm^2^. To induce the proinflammatory M1 phenotype, 10 ng/mL lipopolysaccharide (LPS; Sigma, USA) and 100 ng/mL interferon gamma (IFN‐γ; R&D, USA) were added to the culture medium. The induction was conducted at 37°C with 5% CO_2_ for 12 h. The cells were then washed with phosphate buffered saline (PBS) for three times to completely remove the residue of LPS and IFN‐γ. M1 or untreated macrophage cultures were supplemented with PAM‐activated DMEM at a dose of 5%, 10%, or 30% (100 μL per 5000 cells) for 12 h at 37°C with 5% CO_2_. To fully eliminate the excess PAM‐activated DMEM, the cells were washed three times with PBS. Before collecting the conditioned medium, phenotype‐switched macrophages underwent a 24‐h starvation period. The conditioned medium was labeled as M1CM and M0CM, respectively, depending on whether the macrophages had been treated with or without LPS/IFN‐γ.

### 2.3 Live/dead cell viability assay

The cellular supernatants were removed after 12 h of PAM culture, and the cells were washed with PBS. Each well received 50 μL of PBS that contained 10 μg/mL propidium iodide and 10 μg/mL Hoechst 33342 for a 30‐min incubation period at 37°C. Incubated cells were then scanned using an In‐Cell Analyzer 6500HS (GE Healthcare, USA, 10× objective). Propidium iodide (PI) and Hoechst 33342 staining were identified at a wavelength of 642 nm and 405 nm, respectively. Live/Dead cell viability analysis was performed using IN Carta Image Analysis Software.^10^ Cell viability is calculated as follows: cell count (PI)/cell count (Hoechst 33342) × 100%.

### 2.4 Scanning electron microscope (SEM)

LPS/IFN‐γ stimulated macrophages were cultured with PAM for 12 h and then fixed with 2.5% glutaraldehyde (Sigma, G5882). The fixed samples were postfixed in 1% osmium tetroxide and then processed through dehydration by increasing ethanol concentrations (50%, 70%, 90%, and 100% vol/vol). The samples were finally gold‐coated for observation of the cell morphology using SEM (FESEM, Zeiss Sigma) under 20 kv using SE2 mode.

### 2.5 Osteogenic differentiation of periodontal ligament cells (PDLCs)

Human research ethics approval was obtained from the Office of Research Ethics and Integrity (OREI), Queensland University of Technology (QUT). The PDLCs used in this study were harvested from caries‐free and periodontally healthy premolars extracted for orthodontic treatment purposes (*n* = 6). The periodontal ligament tissues were isolated from the mid‐third of the tooth root surfaces, trimmed to fine pieces, and rinsed with PBS.^4^ The tissue explants were transferred to a T75 flask with DMEM containing 10% FBS and 1% P/S. The culture medium was replaced twice a week and the outgrown cells were passaged after they had reached around 80% confluence. The cells from passages 2 to 4 were used in the following experiments. For osteogenic differentiation of PDLCs, the macrophage‐derived conditioned medium was mixed 1:1 with osteogenic medium (DMEM supplemented with 20% FBS, 1% P/S, 20 mM β‐glycerophosphate, 100 μM ascorbic acid, and 200 nM dexamethasone) to culture PDLCs for 7 days at 37°C and 5% CO_2_.

### 2.6 Enzyme‐linked immunosorbent assay (ELISA)

The concentrations of interleukin 6 (IL‐6), tumor necrosis factor‐α (TNF‐α), and bone morphogenetic protein 2 (BMP‐2) in the conditioned medium was determined by ELISA kits (R&D, Australia). Each well received 50 μL of either M0CM, M1CM, and PAM‐treated M1CM before being incubated for 2 h at room temperature. After incubation, the supernatant of each well was removed, and the well was rinsed with 400 μL washing buffer. After washing thoroughly, 100 μL murine conjugate was added into each well and incubated at room temperature for 2 h, followed by the same washing steps mentioned above. Before adding 100 μL of stop solution, 100 μL of the substrate solution per well was added and incubated for 30 min in the dark. The absorbance at 450 nm was read using a microplate spectrophotometer (Bio‐Rad, USA) with reference absorbance at 540 nm. The concentrations of cytokines were quantified according to the standard curve. The inflammatory cytokines contained in M1CM and PAM‐treated M1CM (10%) were further analyzed with a cytokine array kit (Proteome Profiler Mouse XL Cytokine Array, R&D Systems, ARY028).

### 2.7 Gene expression detection

Using the TRIzol® reagent, total RNA from macrophages or PDLCs was extracted (Life Technologies Pty Ltd., Australia). The purity and quantity of RNA were determined spectrophotometrically using a NanoDrop instrument (Thermo Fisher Scientific). The cDNA was synthesized from 1 μg of total RNA using the SensiFAST™ cDNA Synthesis Kit (Bioline Australia Pty Ltd.). The real‐time quantitative reverse transcription polymerase chain reaction (RT‐qPCR) was performed using a QuantStudio 7 Flex Real‐Time PCR System (Applied Biosystems, Thermo Fisher Scientific) with SYBR Green reagent to detect the expression of M1 macrophage markers such as *TNF‐α* and *IL‐6*, as well as the osteogenesis‐related markers of PDLCs including *Runt‐related transcription factor 2* (*RUNX2*), *cementum protein 1* (*CEMP1*), and *osteopontin* (*OPN*). Relative gene expression was normalized against *GAPDH*. The primers used in this part of the study are listed below (Table 1). All experiments were performed in triplicate.

**TABLE 1:** Primers used for RT‐qPCR.

Target gene	Forward primer	Reverse primer
*TNF‐α*	5′‐ctgaacttcggggtgatcgg‐3′	5′‐ggcttgtcactcgaattttgaga‐5′
*IL‐6*	5′‐atagtccttcctaccccaatttcc‐3′	5′‐gatgaattggatggtcttggtcc‐3′
*RUNX2*	5′‐agggactatggcgtcaaaca‐3′	5′‐ggctcacgtcgctcatctt‐3′
*CEMP1*	5′‐gggcacatcaagcactgacag‐3′	5′‐cccttaggaagtggctgtccag‐3′
*OPN*	5′‐caatgaaagccatgaccacatgg‐3′	5′‐ctcatctgcggcatcaggatactg‐3′
*GAPDH*	5′‐tcagcaatgcctcctgcac‐3′	5′‐tctgggtggcagtgatggc‐3′

### 2.8 Alizarin Red S staining

After 14 days of culture with the macrophage‐derived conditioned medium, the PDLCs were fixed with 4% paraformaldehyde (PFA). The fixed cell samples were incubated with 1% Alizarin Red S (A5533, Sigma–Aldrich) for 20 min and rinsed with distilled water. The dye of each sample was extracted using 300 μL 50% acetic acid, let to sit at room temperature for 30 min, and then neutralized with 10% ammonium hydroxide (320,145, Sigma–Aldrich) with the pH set to 4.1. The solutions were centrifuged at 10,000 RPM for 10 min. Finally, 100 μL supernatant of each sample was transferred into a 96‐well plate and measured at 405 nm using a BIO‐RAD microplate absorbance spectrophotometer.

### 2.9 Western blot

The protein of each sample was collected and suspended in 500 μL RIPA lysis buffer (R0278, Sigma) with protease inhibitor (cOmplete, EDTA‐free, Roche) and phosphatase inhibitor (PhosSTOP, Roche). A total of 20 μg of proteins from each sample were loaded and separated onto SDS‐PAGE gels. A continuous current of 100 mA was used to transfer the isolated proteins onto a nitrocellulose membrane (Pall Corporation) at 4°C. Odyssey blocking buffer (LI‐COR Biosciences) was added to protein‐contained membranes to block the unspecific binding. The membranes were incubated with primary antibodies inducible nitric oxide synthase (iNOS, 1:1000, 13120S, Cell Signaling), C‐C chemokine receptor type 7 (CCR7, 1:1000, ab191575, Abcam), Arginase‐1 (1:1000, 93668S, Cell Signaling), microtubule‐associated proteins 1A/1B light chain 3A/B (LC3A/B, 1:1000, 4108S, Cell Signaling), Autophagy related 5 (ATG5, 1:1000, 12994S, Cell Signaling), protein kinase B (Akt, 1:1000, 4685S, Cell Signaling), phospho‐Akt (p‐Akt, 1:1000, 4060S, Cell Signaling), mammalian target of rapamycin (mTOR, 1:1000, 2972S, Cell Signaling), phospho‐mTOR (p‐mTOR, 1:1000, 5536S, Cell Signaling), BMP‐2 (1:1000, ab214821, Abcam), and α‐Tubulin (1:2000, ab15246, Abcam) overnight at 4°C. Primary antibodies that had not been bound were removed by washing in PBS containing 1% Tween 20 (Bio‐Rad, USA). The membranes were then incubated with anti‐mouse/rabbit fluorescence conjugated secondary antibodies at dilutions of 1:10,000 for 1 h at room temperature. Washing in PBS containing 1% Tween 20 eliminated unbound secondary antibodies. The protein bands were visualized using the Odyssey Infrared Imaging System (LI‐COR Biosciences, USA). The relative intensity of protein bands was quantified using the ImageJ software.

### 2.10 Immunofluorescence (IF) staining

PAM‐treated macrophages were fixed with 2.5% glutaraldehyde for 30 min, permeabilized with 0.1% Triton X‐100 for 10 min, and blocked with 5% BSA for 1 h at room temperature. The cells were then incubated with the mouse polyclonal antibody against CD86 (1:100, sc‐28347, Santa Cruz Biotechnology), the rabbit polyclonal antibody against CD206 (1:200, ab64693, Abcam), and the rabbit monoclonal antibody against iNOS (1:100, 13120S, Cell Signaling) overnight at 4°C. The secondary antibodies used were goat anti‐mouse immunoglobulin G (IgG) Alexa Fluor 488 (1:100, A‐11001, Life Technologies) and goat anti‐rabbit IgG Alexa Fluor 568 (1:200, A‐11036, Life Technologies). Nuclei was labeled by Hoechst 33342 (Thermo Fisher Scientific, USA), while cytoskeleton was labeled with phalloidin (Thermo Fisher Scientific, USA). ProLong Gold Antifade Mountant (Thermo Fisher Scientific, Australia) was used to mount samples on glass slides, and the InCell 6500HS (GE Healthcare, USA, 100× objective) was used to view the images. The intensity of CD86 and CD206 was measured with the ImageJ software.

### 2.11 Monodansylcadaverine staining

The autofluorescent agent monodansylcadaverine (MDC) was used to identify the autophagic vacuoles in PAM‐treated macrophages. Macrophages were stained with 20 μM MDC (Sigma–Aldrich) for 30 min at 37°C in the dark after being fixed with 4% PFA. The cells were then rinsed with PBS and a Nikon A1R confocal microscope was used to identify the MDC staining. The intracellular MDC intensity was quantified by the BIO‐RAD microplate absorbance spectrophotometer at 340 nm.

### 2.12 Statistical analysis

All data were presented as mean ± standard deviation (SD) for three independent experiments. Statistical differences between each group were determined by one‐way ANOVA with Bonferroni's multiple comparison tests. A *p* < 0.05 was considered statistically significant.

## 3 RESULTS

### 3.1 PAM did not affect the growth or viability of macrophages

**FIGURE 1:**
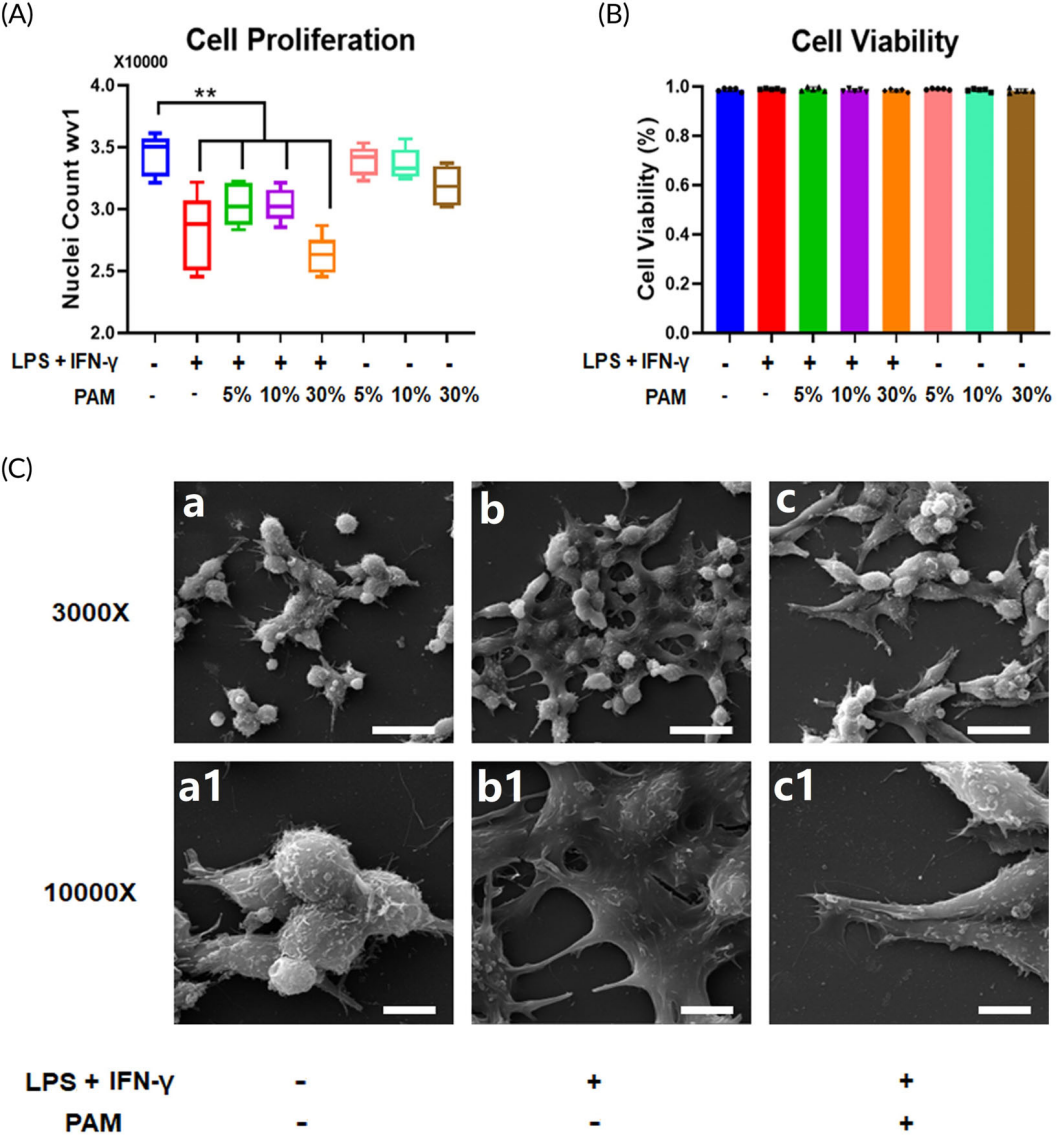
PAM did not affect the growth or viability of macrophages. (A) The average nuclei number of M0/M1 macrophages after PAM treatment is shown on the box plot graph. The results of five independent repeats are presented as the mean ± SD (**p < 0.01, one‐way ANOVA); (B) the average cell viability of M0/M1 macrophages treated with PAM is displayed in a bar graph. Three independent repeats were conducted, and the data are shown as the mean ± SD (*p < 0.05, one‐way ANOVA); Representative SEM images display the morphology changes of M0/M1 macrophages and PAM‐treated M1 macrophages (a, b, and c: low magnification at 3000× and scale bars represented 20 μm; a1, b1, and c1: high magnification at 10,000× and scale bars represented 5 μm).

The number of nuclei indicates the level of cell growth. Murine macrophages were induced towards an M1 subtype by LPS and IFN‐γ stimulation for 12 h. The number of the activated macrophages' nuclei dropped from 34,340 ± 1475 to 28,054 ± 2718 (Figure 1A). However, when the macrophages were treated with PAM at various concentrations (5%, 10%, or 30%), the number of nuclei, which was around 34,000, did not change significantly compared to the M0 subtype (Figure 1A). Additionally, PAM at various concentrations (5%, 10%, or 30%) had no effect on the proliferation of macrophages since, in comparison to M1 macrophages without PAM treatment, the average nuclei number of PAM‐treated macrophages did not vary significantly. Cell viability was measured as cell count (PI)/cell count (Hoechst 33342) × 100%. Our results indicate that the cell viability was not significantly impacted by LPS and IFN‐γ stimulation, and the same pattern was seen in M0 or M1 macrophages cultured in PAM at various concentrations (5%, 10%, or 30%) (Figure 1B). LPS/ IFN‐γ stimulation on macrophages resulted in the extension of dendritic structure (Figure 1Cb,Cb1), while turning to a spindle‐like shape after PAM treatment (Figure 1Cc,Cc1).

### 3.2 PAM reduced the expression of M1 markers in an inflammatory microenvironment and promoted M1 to M2 phenotypic shift of macrophages

**FIGURE 2:**
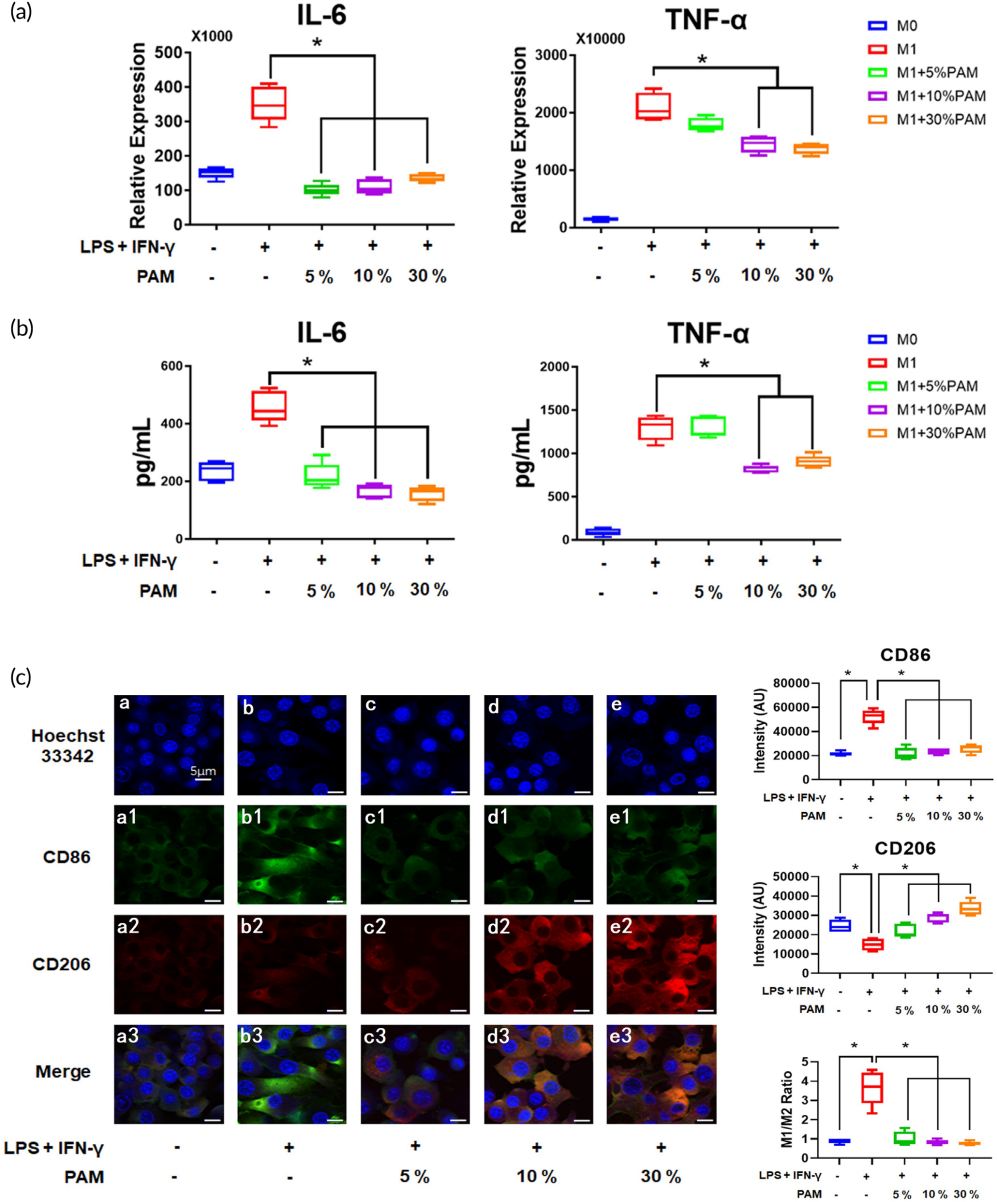
PAM reduced the expression of M1 markers in an inflammatory microenvironment and promoted M1 to M2 phenotypic shift of macrophages. (A) The gene expression levels of inflammatory macrophage markers after PAM treatment were displayed in a box plot graph. The data from five independent experiments were shown as mean ± SD (*p < 0.05, one‐way ANOVA); (B) the concentrations of inflammatory cytokines derived from PAM‐stimulated macrophages are shown. The results of five independent experiments are presented as mean ± SD (*p < 0.05, one‐way ANOVA); (C) the IF staining images show the change of M1/M2 ratio after PAM treatment (scale bars: 5 μm). Data from five randomly selected field of view (FOV) were analyzed using the ImageJ software to determine the intensity of CD86 and CD206. The results are presented as mean ± SD (*p < 0.05, one‐way ANOVA).

The levels of inflammatory markers, such as IL‐6 and TNF‐α elevated dramatically by LPS and IFN‐γ stimulation of macrophages (Figure 2A), whereas PAM treatment resulted in a concentration‐dependent deduction (Figure 2A). ELISA was used to measure the production of proinflammatory cytokines in the cell culture medium, and it corroborated the same trend. Significantly more IL‐6 and TNF‐α were present in the conditioned medium derived from M1 macrophages than from M0 macrophages (Figure 2B). When PAM was used to stimulate macrophages, the levels of IL‐6 and TNF‐α in the conditioned medium were significantly lower than those released by M1 macrophages (Figure 2B). In LPS and IFN‐γ stimulated macrophages, the IF staining revealed an increased intensity of CD86 (M1 macrophage marker) and a decreased intensity of CD206 (M2 macrophage marker) (Figure 2Cb1,Cb2,Cb3), which led to an enhanced M1/M2 ratio and suggested a proinflammatory feature. The high M1/M2 ratio was reversed when macrophages were cultured with PAM (Figure 2Cc3,Cd3,Ce3).

### 3.3 PAM increased the autophagic activity in M1 macrophages

**FIGURE 3:**
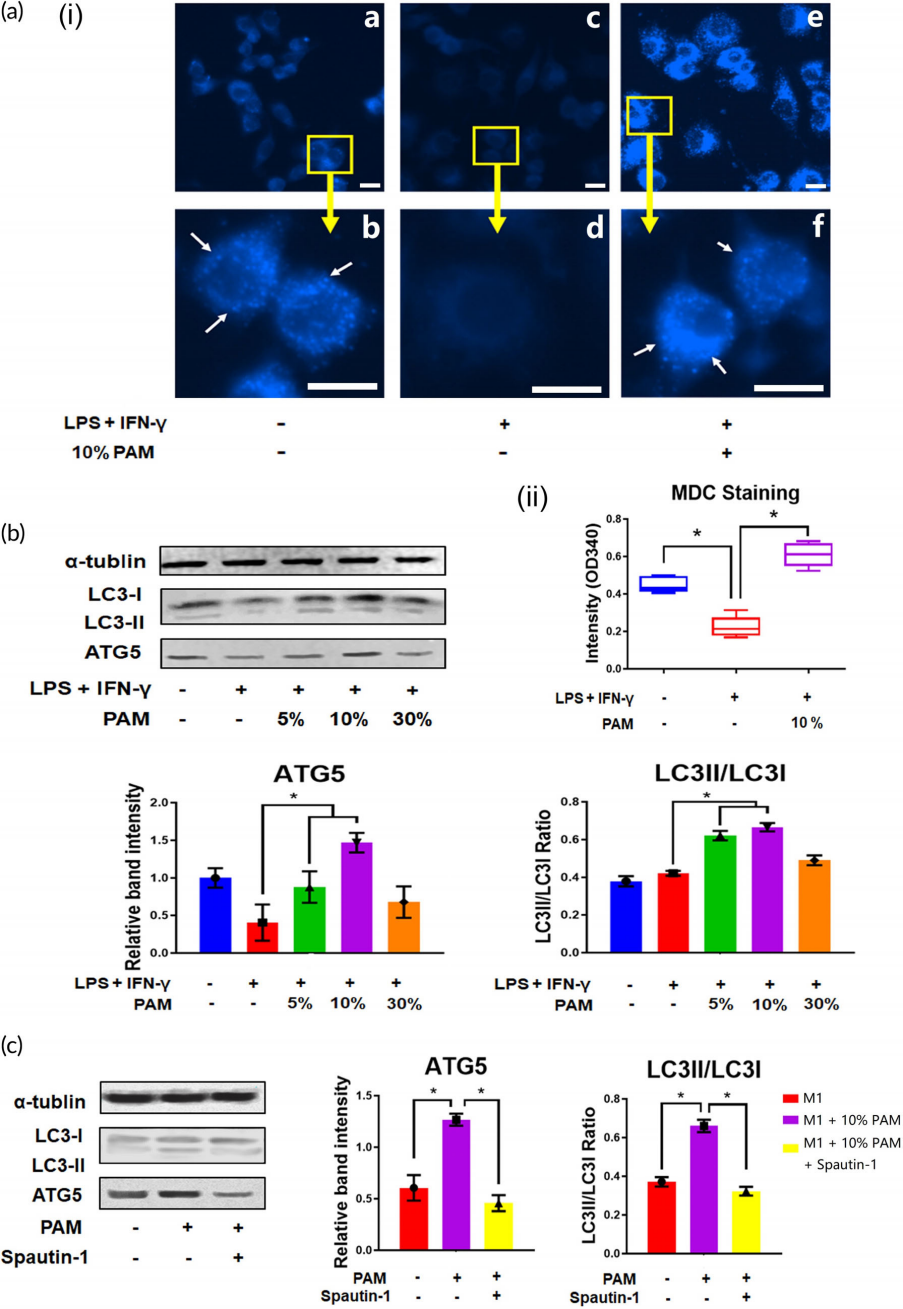
PAM increased the autophagic activity in M1 macrophages. (A) Autophagosomes in PAM‐stimulated macrophages were visible in fluorescence images (autophagosomes: white arrows; scale bar: 10 μm), the intensities of 5 independent experiments are experiments are presented as mean ± SD (*p < 0.05, one‐way ANOVA); (B) Western blotting was used to determine the expression of autophagic markers in PAM‐stimulated macrophages, and the results from three independent experiments are presented in a bar chart as mean ± SD (*p < 0.05, one‐way ANOVA). (C) PAM‐stimulated macrophages underwent changes in autophagic activity after treatment with Spautin‐1 (autophagic inhibitor). The data from three independent experiments are presented as mean ± SD (*p < 0.05, one‐way ANOVA).

Autophagic activity is represented by the quantity of autophagosomes, which can be stained with MDC.^21^ PAM‐treated M1 macrophages exhibited a higher level of intercellular autophagosomes than the M1 macrophages (Figure 3Ai). This was further supported by the higher intensity detected in PAM‐stimulated M1 macrophages than that in M1 macrophages (Figure 3Aii). Western blot was used to detect the intercellular protein markers of autophagic activity, ATG5, and LC3II/LC3I. The relative band intensity demonstrated that both ATG5 and LC3II/LC3I levels in PAM‐treated M1 macrophages were elevated (Figure 3B). Spautin‐1 (10 μM) was added to the PAM‐treated M1 macrophages to deactivate autophagy, which led to a reduction in the expression of ATG5 and LC3II/LC3I (Figure 3C).

### 3.4 PAM inhibited the Akt signaling pathway in macrophages

**FIGURE 4:**
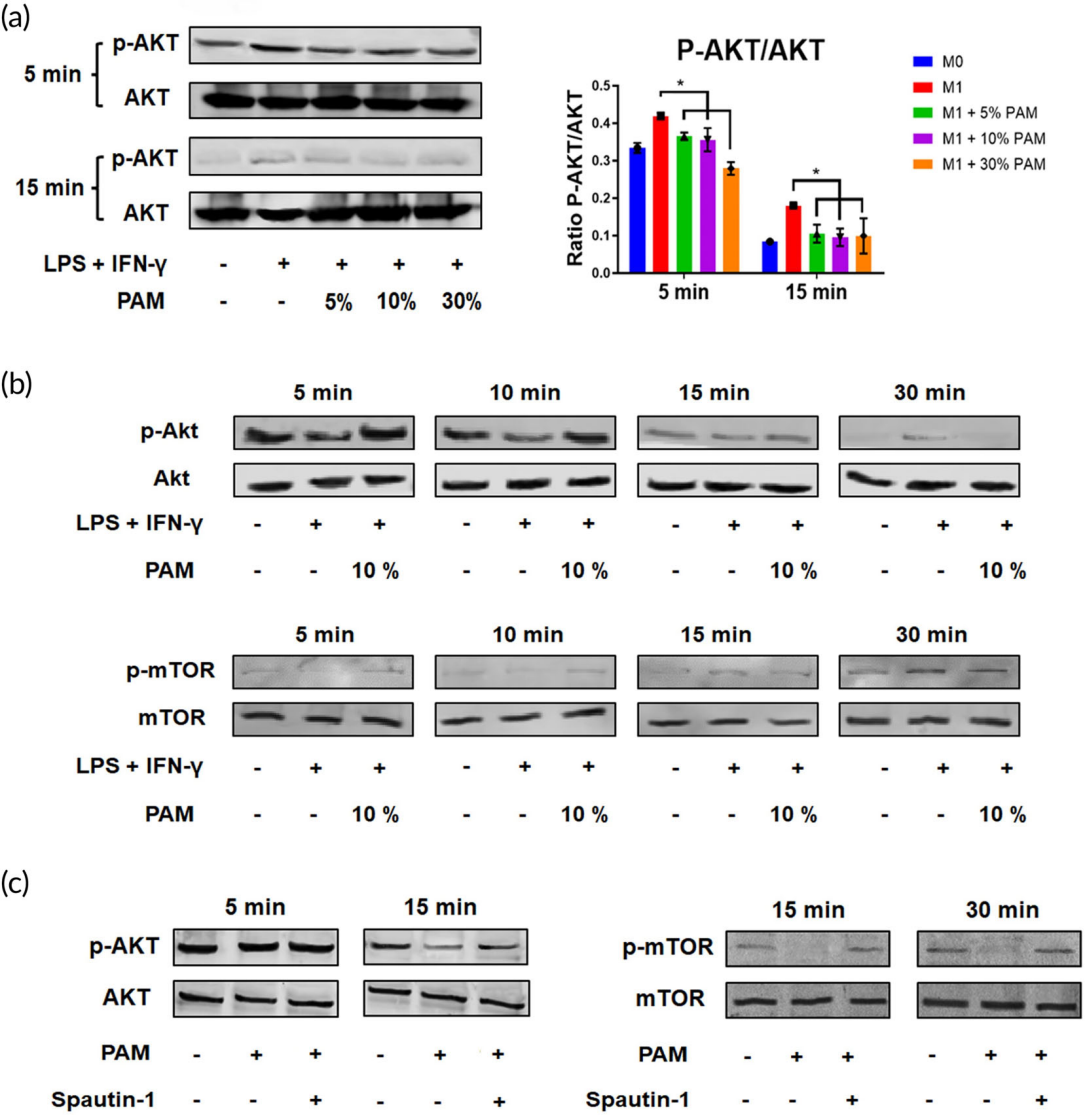
PAM suppressed Akt signaling pathway activation in macrophages. (A) Changes of Akt signaling pathway in PAM‐stimulated macrophages were detected by Western blotting, and the intensities from five independent experiments are shown as mean ± SD (*p < 0.05, one‐way ANOVA); (B) time‐dependent changes in the Akt signaling pathway were observed in PAM‐stimulated macrophages (detected at various time points after PAM treatment: 5 min, 10 min, 15 min, and 30 min); (C) changes of Akt signaling pathway in PAM‐stimulated macrophages after Spautin‐1 treatment in a time‐dependent manner.

One important mechanism that controls autophagic activity is the Akt signaling pathway, the activation status of which may be determined by calculating the ratio of p‐Akt/Akt.^22^, ^23^ After 5 min of PAM stimulation, M1 macrophages showed a substantial increase in the ratio of p‐Akt/Akt (Figure 4A). The Akt signaling pathway remained at a higher level in M1 macrophages, but gradually decreased after 15 min of PAM treatment (Figure 4A). We then looked into the changes in Akt signaling in PAM‐treated M1 macrophages over time and discovered that Akt was active as early as 5 min in M1 macrophages, while mTOR signaling was not activated until 15 min later (Figure 4B). Following 10% PAM stimulation, p‐Akt and p‐mTOR were both deactivated in M1 macrophages (Figure 4B). When Spautin‐1 was administered to deactivate autophagy, the levels of p‐Akt and p‐mTOR were increased (Figure 4C), suggesting autophagy inhibition resulted in an upregulation of the Akt signaling pathway in PAM‐treated M1 macrophages.

### 3.5 PAM‐treated macrophages promoted PDLC mineralization

**FIGURE 5:**
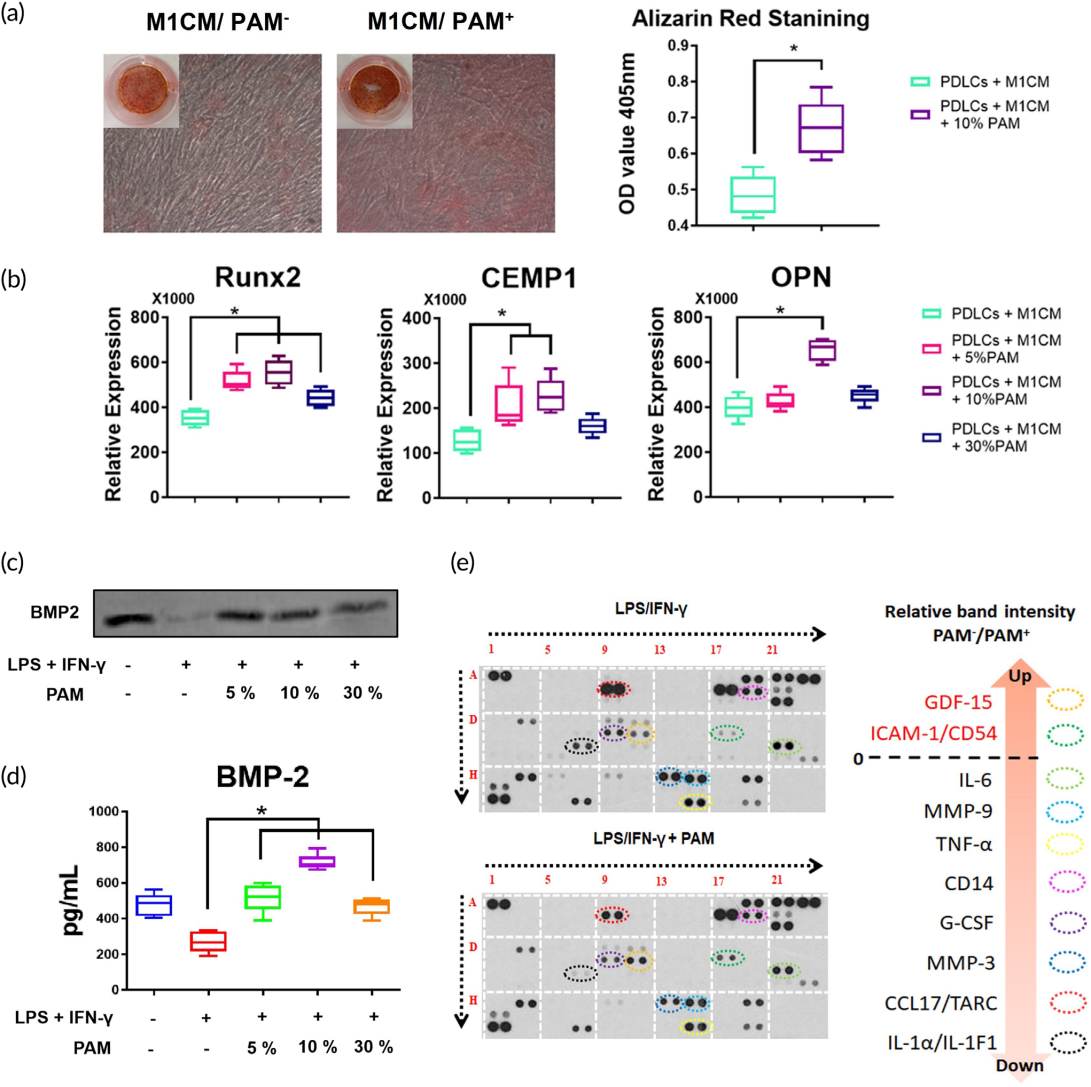
PAM‐treated macrophage promoted PDLC mineralization. (A) Alizarin Red S staining images of PDLCs cultured with PAM‐treated macrophage‐derived conditioned medium. The average optical density (OD) value from five independent repetitions of Alizarin Red S staining is presented on a box plot graph showed as the mean ± SD (*p < 0.05, one‐way ANOVA); (B) The box plot graph displays the osteogenesis‐ and cementogenesis‐related gene expression of PDLCs cultured with PAM‐treated macrophage‐derived conditioned medium. The data from five independent experiments are shown as the mean ± SD (*p < 0.05, one‐way ANOVA). (C) The expression of BMP‐2 in the macrophage‐derived conditioned medium was detected by Western blot; (D) the concentration of BMP‐2 in the macrophage‐derived conditioned medium was measured with ELISA, and data from 5 independent experiments are shown as mean ± SD (*p < 0.05, one‐way ANOVA); (E) various cytokines secreted from M1 macrophages and PAM‐treated M1 macrophages were detected using a cytokine array.

PDLCs were osteogenically differentiated for 7 days with the supplementation of the conditioned medium derived from M1 macrophages with or without PAM treatment. The PDLCs preconditioned with PAM‐stimulated M1 macrophages demonstrated an accelerated mineralization capacity, as evidenced by an increase in the number of mineralization nodules visible under a microscope and an intensification of Alizarin Red S staining (Figure 5A). When PDLCs were cultured in the conditioned medium derived from PAM‐treated M1 macrophages as opposed to the untreated M1 macrophages, Runx2, a critical regulator of osteogenesis,^24^ showed a higher expression (Figure 5B). Under the stimulation of PAM‐treated M1CM, CEMP1, a protein coding gene associated with cementum differentiation,^25^ was upregulated (Figure 5B). Additionally, it was discovered that the expression of OPN, a highly phosphorylated glycoprotein with cell attachment capabilities and a significant component of the bone matrix, was elevated in PDLCs induced by 10% PAM‐treated M1CM (Figure 5B). The secretions of major bone homeostasis related cytokines such as BMP‐2, growth differentiation factor‐15 (GDF‐15), and intercellular cell adhesion molecule‐1 (ICAM‐1) were found significantly increased in the PAM‐treated M1 macrophage‐derived conditioned medium compared to those without PAM treatment (Figure 5C–E).

## 4 DISCUSSION

Macrophage, belonging to innate immune system, is one of the first members response to infectious pathogens, implants, and biomaterials.^26^, ^27^ Recent research has focused on macrophages because of their regulatory property function, which is essential for tissue repair.^28^ Indirectly promoting tissue regeneration via stimulating macrophages is a significant concept of drug discovery, therapy design, and biomaterial synthesis.^26^, ^27^, ^28^ From our observation, M0 and M1 macrophage viability was unaffected by the PAM treatment, despite prior reports that LPS and IFN‐γ stimulation may limit cell proliferation when macrophage polarization towards M1 is induced.^2^ PAM and gaseous plasma produced by an argon plasma jet were characterized using OES, and the discharged compounds (NO, N, N_2_, OH, and O) were found to be enriched (Figure S1). Reactive nitrogen species (RNS) and reactive oxygen species (ROS) were found to be the most stable and bio‐reactive compounds in PAM.^10^, ^15^ High levels of intercellular ROS may force cells to experience more oxidative stress, which lowers cell viability.^29^ Interestingly, even at a concentration of 30% in our present study, the viability of macrophages did not show significant changes when treated with PAM. Our observation may have been caused by a relatively low concentration of RNS/ROS that was present in the PAM for only a brief period of time. The reactivated components contained in PAM aqueous are sustained less than 24 h,^17^, ^30^ so PAM would not further affect the viability of macrophages after the half‐life of ROS and RNS. However, LPS/IFN‐γ stimulation on macrophages resulted in the extension of dendritic structure (Figure 1Cb,Cb1), which resembled the feature of a typical M1 macrophage subtype.^1^, ^3^, ^31^ Morphologic change from a star‐like shape to a spindle‐like shape was observed after applying PAM to M1 macrophages (Figure 1Cc,Cc1), which is normally present by the M2 subtype.^31^ It can be confirmed that, rather than affecting cell viability, PAM tends to cause macrophage subtype switching.

It is generally established that exogenous stimuli such as LPS and IFN‐γ can polarize macrophages towards an M1 subtype.^32^ The efficacy of the stimulation can be demonstrated by the rise in IL‐6 and TNF‐α gene expression and protein production. As each subtype plays a different role during inflammation, and M1 macrophages are in charge of triggering inflammatory responses, therefore it is imperative for macrophages to switch to a proregenerative subtype in a timely manner.^3^, ^33^ PAM stimulation can prevent macrophage polarization towards an M1 subtype, as evidenced by the decreased gene expression and protein production of IL‐6 and TNF‐α in PAM‐treated M1 macrophages. The PAM treatment markedly decreased the protein levels of the M1 macrophage markers iNOS and CCR7, and increased the expression of the M2 macrophage marker Arginase 1, similar to the results of gene expression (Figure S2A). iNOS, a significant enzyme, is crucial in raising cellular NO levels, which increases oxidative stress and aids macrophages in combating pathogens and inducing inflammation.^32^, ^34^ PAM treatment reduced the amount of iNOS in M1 macrophages (Figure S2B), showing that it has anti‐inflammatory properties. ROS and RNS were both elevated in PAM, indicating that the M1 macrophages were surrounded by a reactivated oxygen and nitrogen‐rich environment. It was interesting to note that an oxidative microenvironment deactivated proinflammatory macrophages. Recent research suggests that activated macrophages may be able to detect ROS in their immediate environment and depolarize themselves in order to maintain the balance of M1/M2 subtypes and prevent oxidative damage.^35^ This could account for the observation that PAM, which was rich in ROS and RNS, could prevent macrophage polarization towards an M1 subtype.

Since autophagic activity may suppress the generation of inflammatory cytokines, it plays a role in regulating the polarization of macrophages.^36^, ^37^ Autophagy activation promotes the degradation of nuclear factor kappa B (NF‐κB), thereby limiting NF‐κB mediated proinflammatory macrophage polarization.^38^, ^39^ On the other hand, activation of autophagy promotes M2 polarization of macrophages.^40^ It was clear that the LPS/IFN‐γ‐treated macrophages had reduced autophagic activity, further supporting the idea that they had changed into the proinflammatory M1 subtype. PAM treatment restored the autophagic activity in M1 macrophages, indicating that PAM may prevent the polarization of macrophages towards the M1 subtype. Because autophagy can be induced by oxidative stress, which limits macrophage polarization to the M1 subtype, ROS and RNS found in PAM may play a significant role in limiting macrophage polarization.^41^ Additionally, promoting autophagic activity aids in the reversal of cell aging,^41^ suggesting that PAM has anti‐aging potential. Our findings have demonstrated that PAM increases autophagic activity, preventing macrophages from polarizing towards an M1 subtype. The Akt signaling pathway was then the focus of our investigation into the cellular mechanism because its deactivation would boost autophagic activity and consequently have an impact on macrophage polarization.^23^, ^42^ In M1 macrophages, it was discovered that Akt was modestly increased, which caused the downstream marker mTOR to be upregulated. Since the Akt signaling pathway regulates autophagic activity, increased Akt and mTOR signaling contributed to the decline in autophagic activity in M1 macrophages. By restoring the upregulated Akt and mTOR signaling as well as the downregulated autophagic activity, PAM treatment demonstrated its ability to prevent macrophage polarization towards the M1 subtype by deactivating the Akt signaling pathway.

There is controversy regarding the direction in which cold plasma induces macrophage polarization. In 2019, Kaushik's research demonstrated that stimulating monocytes with μ‐DBD can promote macrophage differentiation and M1 polarization. Activated plasma‐polarized macrophages can inhibit cell viability, EMT, and cancer stemness in glioblastoma multiforme.^43^ In 2022, Trzeciak discovered that M0 resting macrophages treated with CAP exhibited an increase in the M2 phenotype, focusing on the effects and changes of plasma‐induced oxidative stress on macrophages in the melanoma tumor microenvironment.^44^ Different plasma treatment parameters and application scenarios, as shown in Table 2, may produce opposite outcomes. However, the above results confirmed that cold plasma can be a modulator of immune cell activation and can stimulate macrophage polarization through M1/M2 differentiation under various treatment conditions. The use of cold plasma in this project to investigate macrophage polarization in an inflammatory environment is therefore of significant scientific value. The molecular mechanism involving cold plasma and macrophages may become a hot topic of future plasma medicine research.

**TABLE 2:** Plasma‐induced macrophage polarization.

The polarization direction of macrophages	Treated cells	Application	Plasma source	Plasma treatment parameters	Ref.
Proinflammatory M1	Human THP‐1 monocytic model cell	Glioblastoma multiforme (GBM) treatment	μ‐DBD	24 kHz, 0.04 w, 1 L/min (N_2_), 1.2 kV, 2.6 mA, 1 min and 3 min	^43^
A “M0/M2‐like” phenotype	Resting M0 macrophages from human plasma of 10 healthy donors	Melanoma treatment	APPJ MiniJet‐R	2.45 GHz, level 4, 2 L/min (Ar), 30 s, 60 s, 120 s	^44^
Promoted M1 to M2 phenotypic shift in an inflammatory microenvironment	The murine macrophage cell line (RAW264.7)	Periodontal regeneration	kINPen 09 Activated PAM	1.7 MHz, 2–6 kV, 5 L/min (Ar), 10 min	This project

The process of osteogenesis requires the participation of macrophages.^45^ While M2 macrophages produce a proregenerative milieu ideal for periodontal tissue repair and regeneration, inflammatory M1 macrophages inhibit PDLC differentiation and mineralization by secreting a range of proinflammatory cytokines.^46^, ^47^ BMP‐2, a member of the transforming growth factor‐β (TGF‐β) super family, is required in initiating the activation of Wnt signaling pathway that promotes the differentiation of PDLCs.^4^, ^48^, ^49^, ^50^ PAM treatment increased BMP‐2 production in M1 macrophage‐derived conditioned medium (Figure 5C,D), suggesting that PAM has the potential to be used in periodontal therapy as an enhancer for osteogenesis and cementogenesis. Besides BMP‐2, two bone homeostasis related factors, GDF‐15 and ICAM‐1, were found to increase in the inflammatory macrophage‐derived conditioned medium after PAM treatment (Figure 5E). GDF‐15 plays an important role in promoting osteoblast differentiation via inducing Wnt signaling pathway.^51^ ICAM‐1 also participates in the interaction between osteoblasts and osteoclasts, which regulates the balance of bone formation and bone resorption.^52^, ^53^ The increasing expression of bone homeostasis‐related cytokines also suggests the potential of applying PAM in periodontal therapy. However, bacterial infection is the primary cause of periodontitis, and its defense requires the involvement of inflammatory cytokines.^5^ It is possible that PAM inhibits macrophage‐derived inflammatory cytokine secretion and may have a negative effect on bacterial resistance. In fact, the overexpression of tissue‐damaging inflammatory cytokines indicates that the disadvantages outweigh the anti‐bacterial benefits.^4^, ^5^ Other studies have demonstrated the anti‐bacterial property of PAM,^30^ which may increase the efficacy of periodontitis treatments. Because PAM is the liquid form of CAP, it can be used as a mouthwash to deliver charged particles, free radicals, electric fields, and metastable species to macrophages in the periodontium. In addition to inflammatory cytokines, macrophage is one of the major producers of exosomes, which contain regulatory factors and play an important role in tissue regeneration.^54^, ^55^ Close correlations exist between lysosomal‐fusion autophagosomes and membrane‐fusion exosomes.^56^ Therefore, PAM may influence the release of macrophage‐derived exosomes by enhancing autophagic activity, a phenomenon worthy of further investigation. As a potential periodontal therapy candidate, the safety of applying PAM orally should be carefully investigated. There were no observable changes in gene expressions in PDLCs treated with PAM (Figure S3). Furthermore, clinical reports that are currently available showed no damage to oral mucosa when applying CAP orally for bacterial infection and oral cancer treatments.^12^, ^13^, ^57^ Our study has demonstrated that PAM treatment did not affect the morphology and functions of PDLCs (Figure S3), and caused no apoptosis to macrophages (Figure 1B), suggesting that PAM is a safe anti‐inflammatory therapeutic option for periodontitis.

## 5 CONCLUSION

**FIGURE 6:**
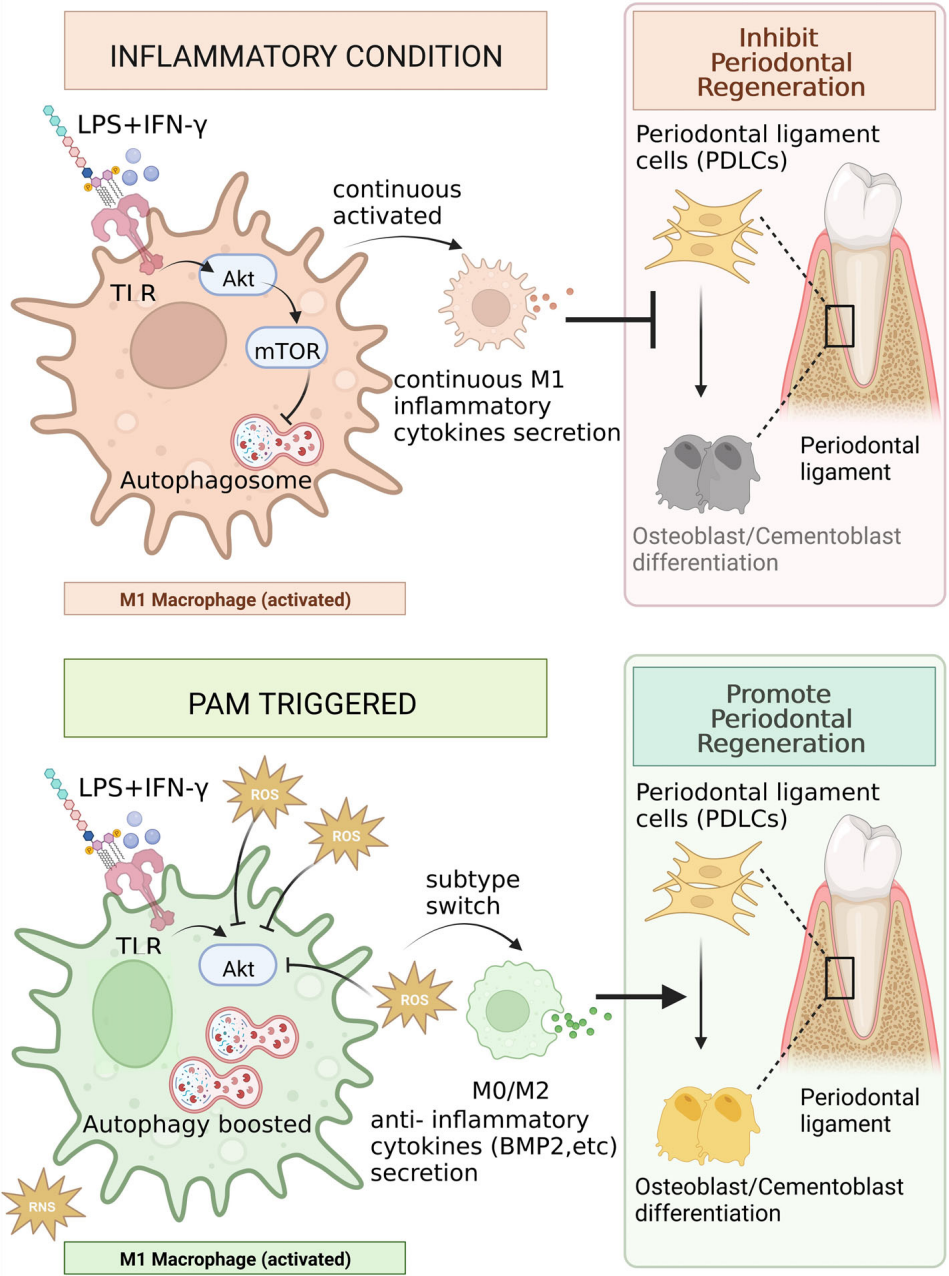
The potential mechanism for PAM‐mediated periodontal regeneration. PAM creates a ROS/RNS enriched microenvironment; High ROS in PAM suppresses the AKT/m‐TOR signaling pathway in the LPS/INF‐γ induced M1 macrophages and boosts the autophagic activities; PAM triggers autophagy activation which results in macrophages switching towards an M2 subtype and secreting anti‐inflammatory cytokines; M2 macrophage‐secreted cytokines, such as BMP‐2, promote periodontal regeneration. Schematic figure created with BioRender.com (access date: the 27th of March 2023).

Plasma activation transforms the culture medium into a microenvironment replete with ROS and RNS. The ROS/RNS boosts autophagic activity by deactivating the Akt signaling pathway, which prevents macrophages from switching towards the proinflammatory M1 subtype. In addition to having anti‐inflammatory properties, PAM also promotes the production of factors associated with osteogenesis and cementogenesis, making it an excellent candidate for periodontal therapeutic applications (Figure 6).

## AUTHOR CONTRIBUTIONS

**Shengfang Wang:** Data curation (equal); formal analysis (lead); investigation (equal); methodology (lead); writing – original draft (equal). **Peiyu Wang:** Data curation (equal); formal analysis (supporting); investigation (equal); methodology (supporting); writing – original draft (equal). **Erik W. Thompson:** Methodology (supporting); resources (supporting); writing – review and editing (supporting). **Kostya (Ken) Ostrikov:** Methodology (supporting); resources (supporting); writing – review and editing (supporting). **Yin Xiao:** Funding acquisition (supporting); project administration (supporting); resources (supporting); supervision (supporting); writing – review and editing (supporting). **Yinghong Zhou:** Conceptualization (lead); formal analysis (lead); funding acquisition (lead); investigation (supporting); methodology (supporting); project administration (equal); supervision (lead); validation (lead); writing – review and editing (lead).

## FUNDING INFORMATION

Open access publishing facilitated by The University of Queensland, as part of the Wiley—The University of Queensland agreement via the Council of Australian University Librarians. WOA Institution: The University of Queensland. Consortia Name: CAUL 2023.

## CONFLICT OF INTEREST STATEMENT

The authors have no conflicts of interest to declare.

## TRANSLATIONAL IMPACT STATEMENT

Plasma‐activated medium (PAM) is enriched with ROS and RNS, leading to an increase in autophagic activity induced by the inactivation of the Akt signaling pathway. This prevents macrophages from switching towards the proinflammatory M1 subtype, and promotes the production of factors associated with bone and cementum formation, making PAM an excellent candidate for periodontal therapeutic applications.

## Supporting information


**Figure S1.** Characterization of PAM. (A) The composition of culture medium treated with the kINPen plasma jet, with the major reactive groups identified by peak labeling (pink area is the medium phase; blue area is the air phase); (B) The process of generation of plasma‐activated medium (schematic figure created with BioRender.com).
**Figure S2.** PAM treatment reduced the expression of M1 macrophage markers while increasing M2 macrophage marker expression. (A) Western blotting was used to determine the protein expression levels of activated macrophage (M1 or M2) markers, and the results from three independent experiments are displayed as mean ± SD (*p < 0.05, one‐way ANOVA); (B) the IF staining images show the changes of intracellular iNOS after PAM treatment (scale bars: 50 μm). Data from five randomly selected field of view (FOV) were analyzed using the ImageJ software to determine the intensity of iNOS. The results are presented as mean ± SD (*p < 0.05, one‐way ANOVA).
**Figure S3.** PAM did not directly affect the mineralization of PDLCs. The box plot graph displayed the osteogenesis‐/cementogenesis‐related gene expression of PDLCs cultured with or without PAM treatment. The data from five independent experiments are shown as the mean ± SD (*p < 0.05, one‐way ANOVA).

## Data Availability

Data available on request from the authors.

## References

[1] Orecchioni M , Ghosheh Y , Pramod AB , Ley K . Macrophage polarization: different gene signatures in M1(LPS+) vs. classically and M2(LPS‐) vs. alternatively activated macrophages. Front Immunol. 2019;10:1084. doi:10.3389/fimmu.2019.01084 31178859 PMC6543837

[2] Wang N , Liang H , Zen K . Molecular mechanisms that influence the macrophage m1‐m2 polarization balance. Front Immunol. 2014;5:614. doi:10.3389/fimmu.2014.00614 25506346 PMC4246889

[3] McCauley J , Bitsaktsis C , Cottrell J . Macrophage subtype and cytokine expression characterization during the acute inflammatory phase of mouse bone fracture repair. J Orthop Res. 2020;38(8):1693‐1702. doi:10.1002/jor.24603 31989683

[4] Zheng X , Wang S , Xiao L , et al. LiCl‐induced immunomodulatory periodontal regeneration via the activation of the Wnt/β‐catenin signaling pathway. J Periodontal Res. 2022;57:835‐848. 35675063 10.1111/jre.13022 PMC9541255

[5] Van Dyke TE , Sima C . Understanding resolution of inflammation in periodontal diseases: is chronic inflammatory periodontitis a failure to resolve? Periodontology 2000. 2020;82(1):205‐213. doi:10.1111/prd.12317 31850636

[6] Dai X , Bazaka K , Richard DJ , Thompson EW , Ostrikov K . The emerging role of gas plasma in oncotherapy. Trends Biotechnol. 2018;36(11):1183‐1198. doi:10.1016/j.tibtech.2018.06.010 30033340

[7] Bekeschus S , Poschkamp B , van der Linde J . Medical gas plasma promotes blood coagulation via platelet activation. Biomaterials. 2021;278:120433. doi:10.1016/j.biomaterials.2020.120433 34562836

[8] Lademann O , Kramer A , Richter H , et al. Skin disinfection by plasma‐tissue interaction: comparison of the effectivity of tissue‐tolerable plasma and a standard antiseptic. Skin Pharmacol Physiol. 2011;24(5):284‐288. doi:10.1159/000329913 21709431

[9] Tavares‐da‐Silva E , Pereira E , Pires AS , et al. Cold atmospheric plasma, a novel approach against bladder cancer, with higher sensitivity for the high‐grade cell line. Biology (Basel). 2021;10(1):41. doi:10.3390/biology10010041 33435434 PMC7828061

[10] Wang P , Zhou R , Thomas P , et al. Epithelial‐to‐mesenchymal transition enhances cancer cell sensitivity to cytotoxic effects of cold atmospheric plasmas in breast and bladder cancer systems. Cancers (Basel). 2021;13(12):2889. doi:10.3390/cancers13122889 34207708 PMC8226878

[11] Xiang L , Xu X , Zhang S , Cai D , Dai X . Cold atmospheric plasma conveys selectivity on triple negative breast cancer cells both in vitro and in vivo. Free Radical Biology and Medicine. 2018;124:205‐213. doi:10.1016/j.freeradbiomed.2018.06.001 29870749

[12] Rupf S , Lehmann A , Hannig M , et al. Killing of adherent oral microbes by a non‐thermal atmospheric plasma jet. J Med Microbiol. 2010;59(Pt 2):206‐212. doi:10.1099/jmm.0.013714-0 19910483

[13] Rutkowski R , Daeschlein G , von Woedtke T , Smeets R , Gosau M , Metelmann H‐R . Long‐term risk assessment for medical application of cold atmospheric pressure plasma. Diagnostics. 2020;10(4):210. doi:10.3390/diagnostics10040210 32290487 PMC7235715

[14] Schuster M , Rutkowski R , Hauschild A , et al. Side effects in cold plasma treatment of advanced oral cancer—clinical data and biological interpretation. Clinical Plasma Medicine. 2018;10:9‐15. doi:10.1016/j.cpme.2018.04.001

[15] Wang P , Zhou R , Zhou R , et al. Cold atmospheric plasma for preventing infection of viruses that use ACE2 for entry. Theranostics. 2022;12(6):2811‐2832. 35401827 10.7150/thno.70098 PMC8965494

[16] Zhou R , Wang P , Guo Y , et al. Prussian blue analogue nanoenzymes mitigate oxidative stress and boost bio‐fermentation. Nanoscale. 2019;11(41):19497‐19505. 31553036 10.1039/c9nr04951g

[17] Zhou R , Zhou R , Wang P , et al. Plasma‐activated water: generation, origin of reactive species and biological applications. J Phys D Appl Phys. 2020;53(30):303001.

[18] Azzariti A , Iacobazzi RM , Di Fonte R , et al. Plasma‐activated medium triggers cell death and the presentation of immune activating danger signals in melanoma and pancreatic cancer cells. Sci Rep. 2019;9(1):4099. doi:10.1038/s41598-019-40637-z 30858524 PMC6411873

[19] Turrini E , Laurita R , Simoncelli E , et al. Plasma‐activated medium as an innovative anticancer strategy: insight into its cellular and molecular impact on in vitro leukemia cells. Plasma Processes Polym. 2020;17(10):2000007. doi:10.1002/ppap.202000007

[20] Reuter S , Von Woedtke T , Weltmann K‐D . The kINPen—a review on physics and chemistry of the atmospheric pressure plasma jet and its applications. J Phys D Appl Phys. 2018;51(23):233001.

[21] Biederbick A , Kern HF , Elsässer HP . Monodansylcadaverine (MDC) is a specific in vivo marker for autophagic vacuoles. Eur J Cell Biol. 1995;66(1):3‐14. 7750517

[22] Manning BD , Cantley LC . AKT/PKB signaling: navigating downstream. Cell. 2007;129(7):1261‐1274. doi:10.1016/j.cell.2007.06.009 17604717 PMC2756685

[23] Wang RC , Wei Y , An Z , et al. Akt‐mediated regulation of autophagy and tumorigenesis through Beclin 1 phosphorylation. Science. 2012;338(6109):956‐959. doi:10.1126/science.1225967 23112296 PMC3507442

[24] Lee K‐S , Kim H‐J , Li Q‐L , et al. Runx2 is a common target of transforming growth factor beta1 and bone morphogenetic protein 2, and cooperation between Runx2 and Smad5 induces osteoblast‐specific gene expression in the pluripotent mesenchymal precursor cell line C2C12. Mol Cell Biol. 2000;20(23):8783‐8792. doi:10.1128/MCB.20.23.8783-8792.2000 11073979 PMC86511

[25] Arzate H , Zeichner‐David M , Mercado‐Celis G . Cementum proteins: role in cementogenesis, biomineralization, periodontium formation and regeneration. Periodontology. 2015;67(1):211‐233. doi:10.1111/prd.12062 25494602

[26] Chen Z , Klein T , Murray RZ , et al. Osteoimmunomodulation for the development of advanced bone biomaterials. Mater Today. 2016;19(6):304‐321. doi:10.1016/j.mattod.2015.11.004

[27] Xu C , Xiao L , Cao Y , et al. Mesoporous silica rods with cone shaped pores modulate inflammation and deliver BMP‐2 for bone regeneration. Nano Res. 2020;13(9):2323‐2331. doi:10.1007/s12274-020-2783-z

[28] Xu C , Dai H , Hosseinpour S , Hua S . Advances in porous inorganic nanomaterials for bone regeneration. Nano TransMed. 2022;1(1):9130005. doi:10.26599/ntm.2022.9130005

[29] Burton GJ , Jauniaux E . Oxidative stress. Best Pract Res Clin Obstet Gynaecol. 2011;25(3):287‐299. doi:10.1016/j.bpobgyn.2010.10.016 21130690 PMC3101336

[30] Kaushik NK , Ghimire B , Li Y , et al. Biological and medical applications of plasma‐activated media, water and solutions. Biol Chem. 2019;400(1):39‐62. doi:10.1515/hsz-2018-0226 30044757

[31] Gao J , Scheenstra MR , van Dijk A , Veldhuizen EJA , Haagsman HP . A new and efficient culture method for porcine bone marrow‐derived M1‐ and M2‐polarized macrophages. Vet Immunol Immunopathol. 2018;200:7‐15. doi:10.1016/j.vetimm.2018.04.002 29776615

[32] Chan ED , Riches DW . IFN‐γ+ LPS induction of iNOS is modulated by ERK, JNK/SAPK, and p38 mapk in a mouse macrophage cell line. Am J Physiol‐Cell Physiol. 2001;280(3):C441‐C450. 11171562 10.1152/ajpcell.2001.280.3.C441

[33] Utomo L , Bastiaansen‐Jenniskens YM , Verhaar JA , van Osch GJ . Cartilage inflammation and degeneration is enhanced by pro‐inflammatory (M1) macrophages in vitro, but not inhibited directly by anti‐inflammatory (M2) macrophages. Osteoarthr Cartil. 2016;24(12):2162‐2170. doi:10.1016/j.joca.2016.07.018 27502245

[34] Galvan‐Pena S , O'Neill LA . Metabolic reprograming in macrophage polarization. Front Immunol. 2014;5:420. doi:10.3389/fimmu.2014.00420 25228902 PMC4151090

[35] Wang P , Geng J , Gao J , et al. Macrophage achieves self‐protection against oxidative stress‐induced ageing through the Mst‐Nrf2 axis. Nat Commun. 2019;10(1):755. doi:10.1038/s41467-019-08680-6 30765703 PMC6376064

[36] Chang CP , Su YC , Lee PH , Lei HY . Targeting NFKB by autophagy to polarize hepatoma‐associated macrophage differentiation. Autophagy. 2013;9(4):619‐621. doi:10.4161/auto.23546 23360732 PMC3627680

[37] Chen P , Cescon M , Bonaldo P . Autophagy‐mediated regulation of macrophages and its applications for cancer. Autophagy. 2014;10(2):192‐200. 24300480 10.4161/auto.26927 PMC5396097

[38] Qing G , Yan P , Qu Z , Liu H , Xiao G . Hsp90 regulates processing of NF‐κB2 p100 involving protection of NF‐κB‐inducing kinase (NIK) from autophagy‐mediated degradation. Cell Res. 2007;17(6):520‐530. doi:10.1038/cr.2007.47 17563756

[39] Wu MY , Lu JH . Autophagy and macrophage functions: inflammatory response and phagocytosis. Cell. 2019;9(1):70. doi:10.3390/cells9010070 PMC7016593 31892110

[40] Liu T , Wang L , Liang P , et al. USP19 suppresses inflammation and promotes M2‐like macrophage polarization by manipulating NLRP3 function via autophagy. Cell Mol Immunol. 2021;18(10):2431‐2442. doi:10.1038/s41423-020-00567-7 33097834 PMC8484569

[41] Perrotta I , Carito V , Russo E , Tripepi S , Aquila S , Donato G . Macrophage autophagy and oxidative stress: an ultrastructural and immunoelectron microscopical study. Oxid Med Cell Longev. 2011;2011:282739. doi:10.1155/2011/282739 21922037 PMC3172980

[42] Vergadi E , Ieronymaki E , Lyroni K , Vaporidi K , Tsatsanis C . Akt signaling pathway in macrophage activation and M1/M2 polarization. J Immunol. 2017;198(3):1006‐1014. doi:10.4049/jimmunol.1601515 28115590

[43] Kaushik NK , Kaushik N , Adhikari M , et al. Preventing the solid cancer progression via release of anticancer‐cytokines in co‐culture with cold plasma‐stimulated macrophages. Cancer. 2019;11(6):842. 10.3390/cancers11060842 PMC6628390 31216715

[44] Trzeciak ER , Zimmer N , Gehringer I , et al. Oxidative stress differentially influences the survival and metabolism of cells in the melanoma microenvironment. Cell. 2022;11(6):930. doi:10.3390/cells11060930 PMC8946823 35326381

[45] Loi F , Córdova LA , Zhang R , et al. The effects of immunomodulation by macrophage subsets on osteogenesis in vitro. Stem Cell Res Ther. 2016;7(1):15. doi:10.1186/s13287-016-0276-5 26801095 PMC4724110

[46] Sato K , Takayanagi H . Osteoclasts, rheumatoid arthritis, and osteoimmunology. Curr Opin Rheumatol. 2006;18(4):419‐426. 16763464 10.1097/01.bor.0000231912.24740.a5

[47] Zheng X , Huang W . Effects of inflammatory microenvironment mediated by macrophage on the proliferation and osteogenic differentiation of periodontal ligament cells. J Prevent Treat Stomatol Dis. 2018;26(5):297‐303. doi:10.12016/j.issn.2096-1456.2018.05.004

[48] Zhou Y , Lin J , Shao J , et al. Aberrant activation of Wnt signaling pathway altered osteocyte mineralization. Bone. 2019;127:324‐333. doi:10.1016/j.bone.2019.06.027 31260814

[49] Li S , Shao J , Zhou Y , et al. The impact of Wnt signalling and hypoxia on osteogenic and cementogenic differentiation in human periodontal ligament cells. Mol Med Rep. 2016;14(6):4975‐4982. doi:10.3892/mmr.2016.5909 27840938 PMC5355726

[50] Wei F , Zhou Y , Wang J , Liu C , Xiao Y . The immunomodulatory role of BMP‐2 on macrophages to accelerate osteogenesis. Tissue Eng Part A. 2018;24(7–8):584‐594. 28726579 10.1089/ten.TEA.2017.0232

[51] Griner SE , Joshi JP , Nahta R . Growth differentiation factor 15 stimulates rapamycin‐sensitive ovarian cancer cell growth and invasion. Biochem Pharmacol. 2013;85(1):46‐58. doi:10.1016/j.bcp.2012.10.007 23085437 PMC4329765

[52] Tanaka Y , Morimoto I , Nakano Y , et al. Osteoblasts are regulated by the cellular adhesion through ICAM‐1 and VCAM‐1. J Bone Miner Res. 1995;10(10):1462‐1469. 8686501 10.1002/jbmr.5650101006

[53] Tanaka Y , Nakayamada S , Okada Y . Osteoblasts and osteoclasts in bone remodeling and inflammation. Curr Drug Targets‐Inflammation Allergy. 2005;4(3):325‐328. 10.2174/1568010054022015 16101541

[54] Gutierrez‐Vazquez C , Villarroya‐Beltri C , Mittelbrunn M , Sanchez‐Madrid F . Transfer of extracellular vesicles during immune cell‐cell interactions. Immunol Rev. 2013;251(1):125‐142. doi:10.1111/imr.12013 23278745 PMC3740495

[55] Wang S , Yang Y , Li S , Chen H , Zhao Y , Mu J . Recent advances in macrophage‐derived exosomes as delivery vehicles. Nano TransMed. 2022;1(2–4):e9130013. doi:10.26599/ntm.2022.9130013

[56] Xu J , Camfield R , Gorski SM . The interplay between exosomes and autophagy – partners in crime. J Cell Sci. 2018;131(15) jcs215210. doi:10.1242/jcs.215210 30076239

[57] Liu D , Xiong Z , Du T , Zhou X , Cao Y , Lu X . Bacterial‐killing effect of atmospheric pressure non‐equilibrium plasma jet and oral mucosa response. J Huazhong Univ Sci Technolog Med Sci. 2011;31(6):852‐856. doi:10.1007/s11596-011-0690-y 22173512

