# Supplementary material for: Plasma‐activated medium triggers immunomodulation and autophagic activity for periodontal regeneration
Source: Bioeng Transl Med. 2023 May 4;8(4):e10528. doi: 10.1002/btm2.10528 (PMC10354773; doi:10.1002/btm2.10528)
Supplement: Supplementary file 1 — Figure S1. Characterization of PAM. (A) The composition of culture medium treated with the kINPen plasma jet, with the major reactive groups identified by peak labeling (pink area is the medium phase; blue area is the air phase); (B) The process of generation of plasma‐activated medium (schematic figure created with BioRender.com). Figure S2. PAM treatment reduced the expression of M1 macrophage markers while increasing M2 macrophage marker expression. (A) Western blotting was used to determine the protein expression levels of activated macrophage (M1 or M2) markers, and the results from three independent experiments are displayed as mean ± SD (*p < 0.05, one‐way ANOVA); (B) the IF staining images show the changes of intracellular iNOS after PAM treatment (scale bars: 50 μm). Data from five randomly selected field of view (FOV) were analyzed using the ImageJ software to determine the intensity of iNOS. The results are presented as mean ± SD (*p < 0.05, one‐way ANOVA). Figure S3. PAM did not directly affect the mineralization of PDLCs. The box plot graph displayed the osteogenesis‐/cementogenesis‐related gene expression of PDLCs cultured with or without PAM treatment. The data from five independent experiments are shown as the mean ± SD (*p < 0.05, one‐way ANOVA). [file BTM2-8-e10528-s001.docx]

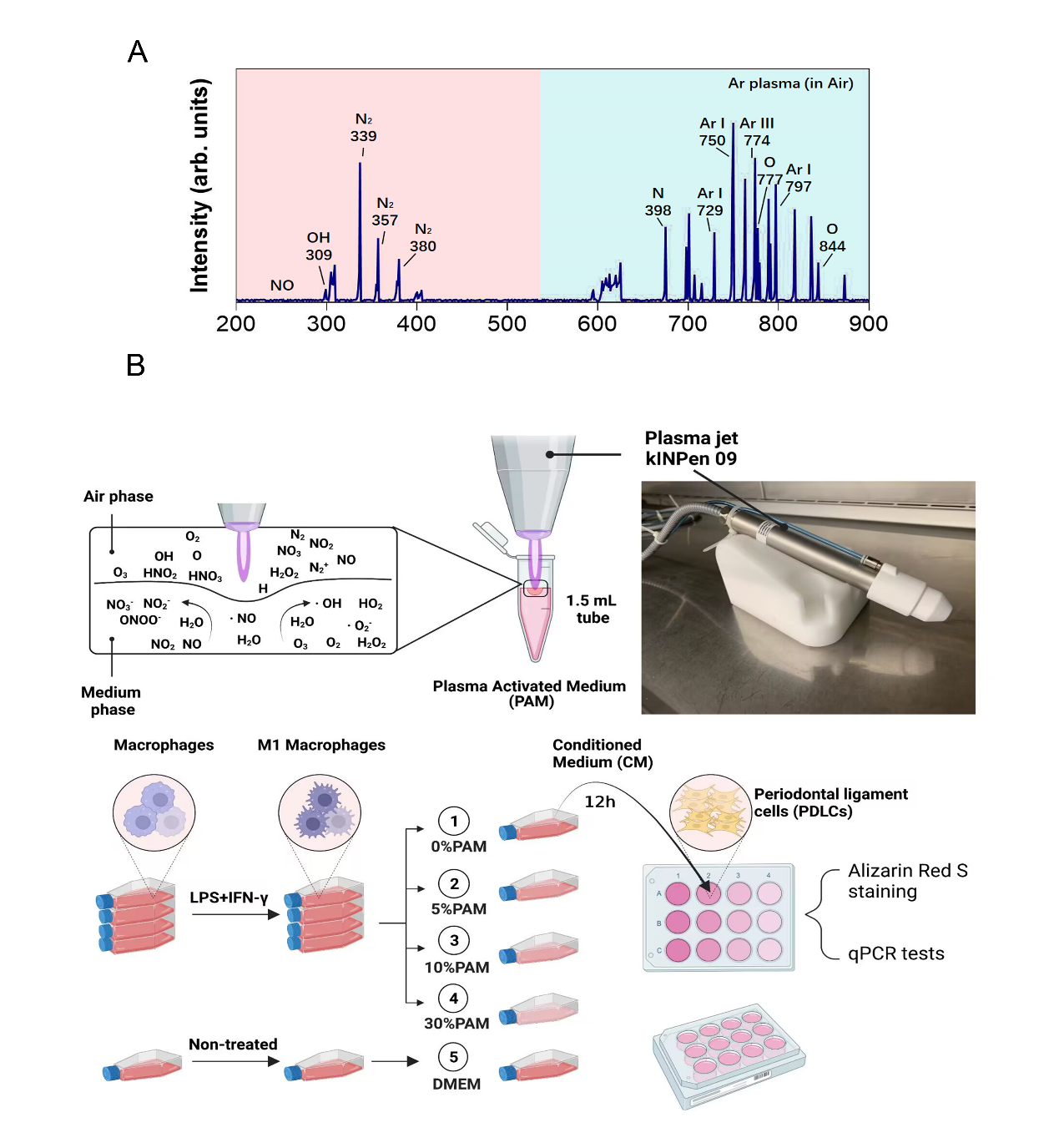


**Supplementary Figure 1. Characterization of PAM.** A: The composition of culture medium treated with the kINPen plasma jet, with the major reactive groups identified by peak labeling (pink area is the medium phase; blue area is the air phase); B: The process of generation of plasma-activated medium (Schematic figure created with BioRender.com).

**
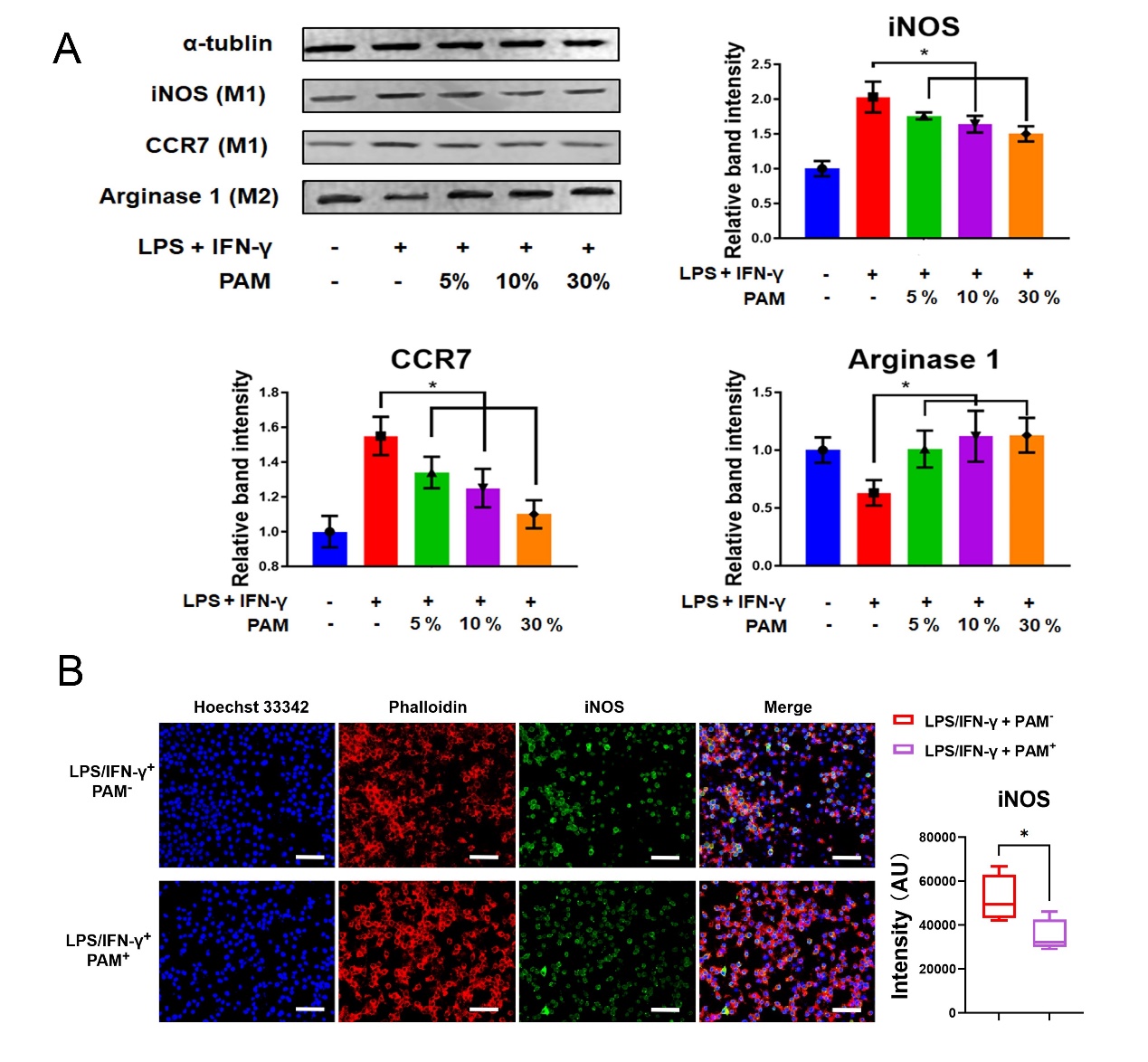
**

**Supplementary Figure 2. PAM treatment reduced the expression of M1 macrophage markers while increasing M2 macrophage marker expression.** A: Western blotting was used to determine the protein expression levels of activated macrophage (M1 or M2) markers, and the results from three independent experiments are displayed as mean±SD (**p*<0.05, one-way ANOVA); B: The IF staining images show the changes of intracellular iNOS after PAM treatment (scale bars: 50 μm). Data from five randomly selected field of view (FOV) were analyzed using ImageJ software to determine the intensity of iNOS. The results are presented as mean±SD (**p*<0.05, one-way ANOVA).


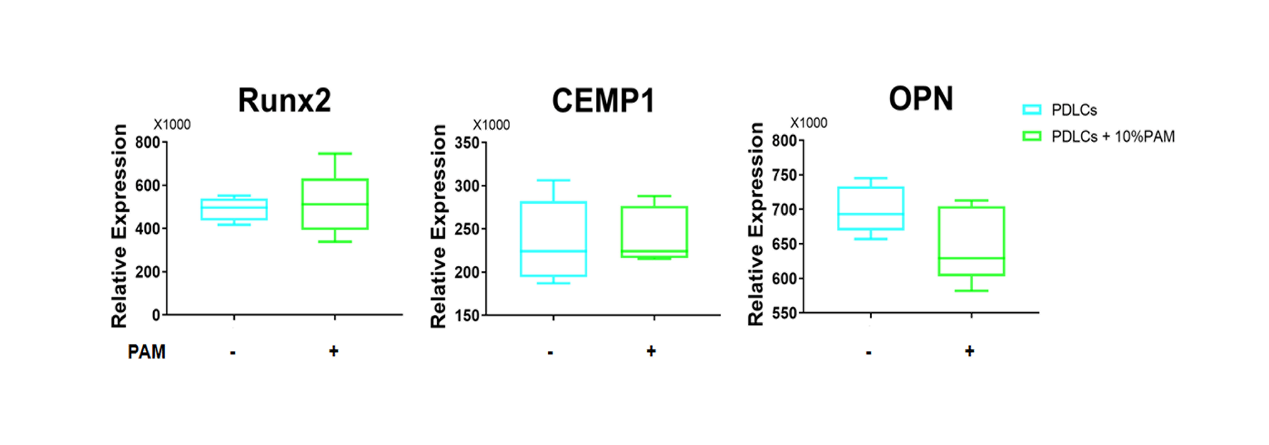


**Supplementary Figure 3. PAM did not directly affect the mineralization of PDLCs.** The box plot graph displayed the osteogenesis-/cementogenesis-related gene expression of PDLCs cultured with or without PAM treatment. The data from five independent experiments are shown as the mean±SD (**p*<0.05, one-way ANOVA).
